# Impact Factor is Off the Ventilator: Survives and is Thriving

**DOI:** 10.12669/pjms.346.16652

**Published:** 2018

**Authors:** Shaukat Ali Jawaid, Masood Jawaid

**Affiliations:** 1 *Shaukat Ali Jawaid Chief Editor, Pakistan Journal of Medical Sciences, Karachi - Pakistan*; 2 *Masood Jawaid Associate Editor Pakistan Journal of Medical Sciences, Karachi - Pakistan*

Impact Factor (IF) of academic journals continues to gain further importance with not only science community but also academic institutions, funding agencies as well as the Editors of academic journals the world over with every passing day. Despite lot of criticism IF has attracted over the last decade in particular and lot of drawbacks, it is still considered as the best available Scientometrics to judge the standard of an academic journal.

It may be mentioned here that Impact Factor is a citation based metrics based on the average number of times an article published in a particular journal has been cited by authors in other journals.^1^ It was developed by Eugene Garfield of Institute of Scientific Information (ISI) of United States in 1960s. IF is calculated by counting the number of citations to articles published in a journal in the last two years by using the following formula:


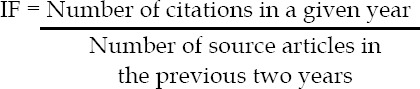


ISI has about 34,171 journals published in academic disciplines which includes 12,271 journals covering medical and social sciences.^2^ One of the major criticism against IF is that any citation in the journals which are not covered by its database are not included. Not only that the ISI’s criteria of inclusion, it is alleged is also not transparent. However, its worst critics also agree that Journal Impact Factor is not all bad and it is not going away any sooner.^3^ We also believe that Impact Factor though important but it is not and should not be the only criteria to judge the standard of a journal 4

Faced with this new situation the Editors and Publishers have started using different strategies to increase their Impact Factor i.e by refusing to publish supplements, encouraging authors and reviewers to cite papers published in their journals. Ensure early publication of papers which are likely to be cited early in the year and making the papers electronically accessible through their websites as early as possible.^5^ Editors are also not entertaining case reports and other papers which does not have a chance of any citation. Publication of Guidelines by various Expert Groups is preferred as it has potential chance of more citations. Importance of IF has also given birth to Citation Stacking which is defined as “When authors try to boost the citation of their own paper. On detection of citation stacking, Thomson Reuters suspended the Impact Factor of four Brazilian Journals. Appeal by these journals against this decision was turned down by ISI.^6^ Now some journals in Pakistan are also reported to be directing authors and their reviewers to cite papers published in their journal to enhance their Impact Factor. Hence, as stated earlier, Journal Impact Factor retains its Impact and it will not go away soon.^7^ On the other hand World Association of Medical Editors (WAME) also believes that journal editors should look beyond Impact Factor on other indicators like journal visibility, circulation, readership, number of manuscripts it receives and publishes every year in addition to distribution of citations.^8^

San Francisco Declaration on Research Assessment (DORA) issued in December 2012 during the American Society of Biology meeting was considered almost a fatal blow to Impact Factor. DORA emphasized to stop the use of “Journal Impact Factor” in judging the scientists’ work.^9^ It also stated that it should not be used for hiring, promotion of funding decisions.^9^ Brue Albert Editor-in-Chief of SCIENCE which had supported DORA in an editorial commented that “DORA recommendations are critical for keeping science healthy.” 10 Sultan Ayoub Moe commenting on this wrote that “Impact factor went on ventilator: Neither died nor buried”.^11^ But time has proved that Impact Factor is now off the ventilator. It has not only survived but is thriving. The Number of medical journals with Impact Factor from different Muslim countries and other countries in this Region as given in Table-1.^12^,^13^

**Table-I:** Number of Journals with Impact Factor in different countries in the Region

1.	China	203
2.	India	104
3.	Iran	42
4.	Pakistan	12
5.	Saudi Arabia	12
6.	Egypt	6
7.	Kuwait	4
8.	Bangladesh	4
9.	UAE	1(38)*

Qatar, Bahrain, Oman, Morroco, Algeria, Sudan, Tunisia has no journal with IF.

* These 38 journals in UAE are published by an International Publishing Institute Banthan Science Publishing Limited with offices all over the world.

The increasing importance of Impact Factor is evident from the fact that many publishers and Editors who fail to get indexed by Web of Sciences feel proud to decorate the title of their journals with various fake Impact Factors. Some of these commonly known fake IF include the following:

**Table:** 

1. Universal Impact Factor	UIF
2. Global Impact Factor	GIF
3. Current Impact Factor	CIF
4. Scientific Impact Factor	SIF
5. CiteFactor	CiteFactor
6. Unofficial Impact Factor	UOIF

The twelve academic journals from Pakistan which includes three medical journals which enjoy Impact Factor are shown in Table-II

**Table-II:** Pakistani Journals with Impact Factor

No.	Full Journal Title	Total Cites	Journal Impact Factor
1	PAKISTAN VETERINARY JOURNAL	1,098	1.217
2	INTERNATIONAL JOURNAL OF AGRICULTURE AND BIOLOGY	2,304	0.869
3	PAKISTAN JOURNAL OF PHARMACEUTICAL SCIENCES	1,821	0.804
4	INTERNATIONAL JOURNAL OF PHARMACOLOGY	722	0.765
5	PAKISTAN JOURNAL OF BOTANY	4,073	0.750
**6**	**PAKISTAN JOURNAL OF MEDICAL SCIENCES**	**1,602**	**0.719**
**7**	**JOURNAL OF THE PAKISTAN MEDICAL ASSOCIATION**	**2,829**	**0.718**
8	PAKISTAN JOURNAL OF AGRICULTURAL SCIENCES	725	0.677
9	PAKISTAN JOURNAL OF ZOOLOGY	1,175	0.547
**10**	**JCPSP-JOURNAL OF THE COLLEGE OF PHYSICIANS AND SURGEONS PAKISTAN**	**1,488**	**0.439**
11	JOURNAL OF ANIMAL AND PLANT SCIENCES	1,198	0.407
12	JOURNAL OF THE CHEMICAL SOCIETY OF PAKISTAN	747	0.280

***Source:*** Clarivate Analytics: Impact Factor of Pakistani Journals indexed in ISI-Web of Science 2018.

While the editors should concentrate on improving the quality of the manuscripts they accept for publication and accelerate their efforts to improve the standard of the journal, manipulating and artificially boosting their Impact Factor is highly unethical. They should follow author friendly policy, help and guide them to improve their manuscripts with the help of members of the editorial board and peer reviewers. It is nice for the editors to know their rights but they must also remember their duties and responsibilities which include teaching and training through workshops. Many journal editors and bodies of Editors including World Association of Medical Editors (WAME), Eastern Mediterranean Association of Medical Editors (EMAME), Asia-Pacific Association of Medical Editors (APAME), Pakistan Association of Medical Editors (PAME) and editor’s bodies in many other countries are already doing this by organizing workshops and these efforts should not only be continued but also accelerated in collaboration with other professional specialty organizations and medical institutions.
